# NLO+NLL squark and gluino production cross sections with threshold-improved parton distributions

**DOI:** 10.1140/epjc/s10052-016-3892-4

**Published:** 2016-01-28

**Authors:** Wim Beenakker, Christoph Borschensky, Michael Krämer, Anna Kulesza, Eric Laenen, Simone Marzani, Juan Rojo

**Affiliations:** 1 Theoretical High Energy Physics, IMAPP, Faculty of Science, Radboud University Nijmegen, Mailbox 79, P.O. Box 9010, 6500 GL Nijmegen, The Netherlands; 2 Institute of Physics, University of Amsterdam, Amsterdam, The Netherlands; 3 Institute for Theoretical Physics, WWU Münster, 48149 Münster, Germany; 4 Institute for Theoretical Particle Physics and Cosmology, RWTH Aachen University, 52056 Aachen, Germany; 5 ITFA, University of Amsterdam, Science Park 904, 1018 XE Amsterdam, The Netherlands; 6 ITF, Utrecht University, Leuvenlaan 4, 3584 CE Utrecht, The Netherlands; 7 Nikhef Theory Group, Science Park 105, 1098 XG Amsterdam, The Netherlands; 8 Department of Physics, University at Buffalo, The State University of New York, Buffalo, NY 14260-1500 USA; 9 Rudolf Peierls Centre for Theoretical Physics, University of Oxford, 1 Keble Road, Oxford, OX1 3NP UK

## Abstract

We present updated predictions for the cross sections for pair production of squarks and gluinos at the LHC Run II. First of all, we update the calculations based on NLO+NLL partonic cross sections by using the NNPDF3.0NLO global analysis. This study includes a full characterization of theoretical uncertainties from higher orders, PDFs and the strong coupling. Then we explore the implications for this calculation of the recent NNPDF3.0 PDFs with NLO+NLL threshold resummation. We find that the shift in the results induced by the threshold-improved PDFs is within the total theory uncertainty band of the calculation based on NLO PDFs. However, we also observe that the central values of the NLO+NLL cross sections are modified both in a qualitative and a quantitative way, illustrating the relevance and impact of using threshold-improved PDFs together with resummed partonic cross sections. The updated NLO+NLL cross sections based on NNPDF3.0NLO are publicly available in the NLL-fast format, and should be an important ingredient for the interpretation of the searches for supersymmetric particles at Run II.

## Introduction and motivation

Supersymmetry (SUSY) [1–6] is one of the better motivated extensions of the Standard Model (SM). In addition to offering a natural solution to the hierarchy problem, it provides a number of dark-matter candidates and leads to the unification of gauge couplings at high scales. While so far searches for supersymmetry at the LHC 8 TeV have returned null results [7–9], the recently started Run II of the LHC, with its increase in center-of-mass energy and luminosity, opens a wide new region of the SUSY parameter space for scrutiny, extending in particular into the high-mass regime up to 2.5 TeV particles [10]. For this reason, it is crucial to provide precise theory predictions for the cross sections of supersymmetric particle pair production at 13 TeV with a robust estimate of the associated theory uncertainties.

The next-to-leading order (NLO) QCD corrections to the total cross sections for squark and gluino pair production were first computed in [11–13]. Recently, a considerable effort has been made in automating these calculations [14–16], including the decays [17] and matching the NLO corrections with a parton shower [18, 19]. Furthermore, NLO electroweak corrections have also been calculated [20–28]. A significant contribution to the QCD NLO corrections originates from soft-gluon emissions which dominate the region near the production threshold. In this region, the partonic center-of-mass energy $\hat{s}$ is close to the kinematic restriction for the on-shell production of these particles, i.e. $\hat{s} \ge 4m^2$, with *m* being the average mass of the two produced particles. Soft-gluon corrections to squark and gluino pair production can be taken into account to all orders in perturbation theory either using threshold-resummation techniques in Mellin space [29–32] or in the framework of effective theories [33, 34].

Resummation of threshold corrections at the next-to-leading logarithmic (NLL) accuracy has been performed for all processes contributing to squark and gluino production, resulting in matched NLO+NLL predictions [35–41]. Additionally, Coulomb corrections have been resummed simultaneously with soft-gluon corrections in [34, 42]. The progress towards resummation at next-to-next-to-leading logarithmic (NNLL) accuracy and towards the corresponding LHC predictions has been reported in [43–52]. Finite width and bound state effects have been studied for squark and gluino production processes in [53–58]. Resummed calculations for sleptons and gauginos are also available at NLO+NLL accuracy [59–61].

A potential source of theory uncertainty in these calculations arises from the fact that they are based on parton distribution functions (PDFs) extracted from experimental data using partonic cross sections computed at fixed-order, NLO accuracy. Ideally, one should use threshold-improved PDFs determined using the same NLO+NLL theory as that used in the computation of the supersymmetric partonic cross sections. Indeed, from general considerations, one expects that resummed PDFs should partially cancel the typical enhancements found in NLO+NLL partonic cross sections.

Therefore, it is important to ascertain the effects of using threshold-resummed PDFs and quantify their phenomenological relevance for NLO+NLL SUSY cross sections. From the practical point of view, it is essential to determine if the shift induced by the threshold-improved PDFs lies within the estimated theory uncertainty bands, the latter obtained from cross sections based on fixed-order NLO PDFs. If such a shift is larger than these uncertainty bands, it would affect the existing SUSY exclusion limits drawn from the LHC measurements. Until recently, no threshold-improved PDF sets were available.

With the motivation of being able to perform, for the first time NLO+NLL and NNLO+NNLL threshold-resummed calculations together with resummed PDFs, variants of the NNPDF3.0 analysis [62] have now become available [63]. These resummed PDFs differ from their fixed-order counterparts due to the different theory inputs used in the two cases. While in the $\overline{\mathrm{MS}}$ scheme the DGLAP evolution kernels are unaffected by threshold resummation [64], the partonic cross sections for the processes that enter the PDF fit are modified (for example for deep-inelastic structure functions and Drell–Yan rapidity distributions). Therefore, these differences in the partonic cross sections used in the fit (NLO+NLL in the former case, NLO in the latter) translate into differences in the extracted PDFs at the input fitting scale $Q_0$. Once these boundary conditions are determined from the fit, standard DGLAP evolution can be used to evolve both the fixed-order and the resummed PDFs for any other value of $Q^2\ge Q_0^2$.

A limitation of these threshold-improved NNPDF sets is that threshold-resummed calculations are not readily available for all the processes included in the NNPDF3.0 global analysis, in particular for inclusive jets and charged current Drell–Yan production. This implies that the resummed PDFs of [63] are based on a more limited dataset than the global fit, and thus they are affected by somewhat larger uncertainties, in particular for the gluon PDF at large *x*. Therefore, it is necessary to devise a prescription to combine the cross sections from the global, fixed-order, PDF fit, with those of the resummed PDF fit based on the reduced dataset.

The first motivation of the current work is to update the NLO+NLL calculations of squark and gluino pair production at the LHC 13 TeV from Refs. [40, 41] with the NNPDF3.0 NLO global PDF set [62]. While previous NLO+NLL predictions were obtained using older global fits, CTEQ6.6 [65] and MSTW08 [66], the new results using NNPDF3.0 should provide a more accurate estimate of theory uncertainties especially in the high-mass region, where the flexible NNPDF parametrization [67–71] minimizes the possible introduction of theory biases that might arise in fits with fixed functional forms. The NNPDF3.0 analysis also includes a variety of recent measurements, in particular from the LHC Run I, which provide better control on the large-*x* PDF uncertainties. Moreover, the NNPDF3.0 fit is based on an extensive improvement of the positivity constraints to ensure that, even if PDFs might become negative, physical cross sections should be positive, resulting in more reliable PDFs in the very large-*x* region as compared to the previous NNPDF2.3 set [72].

The updated NLO+NLL squark and gluino cross sections computed with NNPDF3.0 NLO are available in the NLL-fast format of fast interpolation tables. They include a complete characterization of theoretical uncertainties from PDFs, higher orders, and the strong coupling $\alpha _s$. The flexible NLL-fast format ensures that the results of this work can be readily used by the ATLAS and CMS collaborations for their interpretation of their SUSY searches.

Subsequently, we present an exploration of the implications of the NNPDF threshold-improved PDFs on the NLO+NLL calculations of supersymmetric particle pair production. As we will show, the result of the calculation where the effects of threshold resummation are included both at the level of PDFs and at the level of partonic cross sections can differ substantially from the calculation where resummation is only applied to partonic cross sections. Reassuringly, the cross sections obtained with the threshold-improved PDFs are contained within the theory uncertainty band (including higher orders and PDF uncertainties) of the traditional calculation based on the fixed-order NNPDF3.0 NLO PDF set. This result constitutes a non-trivial validation of the robustness of current estimates of the total theory uncertainty in SUSY production cross sections. While these differences, therefore, do not affect present exclusion limits, they would become crucial in the case of the discovery of supersymmetric particles, since they would affect the determination of their properties from the LHC data, in the same way as in the extraction of Higgs couplings and branching fractions.

The outline of this paper is as follows. In Sect. 2 we present the update of the NLO+NLL cross sections for squark and gluino pair production at 13 TeV based on the NNPDF3.0 NLO global fit. In Sect. 3 we quantify the impact of threshold-resummed PDFs for the NLO+NLL supersymmetric cross sections. In Sect. 4 we conclude, outline possible future developments and present the delivery of the results of this work. Appendix A discusses an alternative to incorporate threshold-resummation effects into the PDFs.

## NLO+NLL cross sections at 13 TeV with NNPDF3.0NLO

In this section we update the NLO+NLL predictions for squark and gluino pair production cross sections at 13 TeV from Refs. [40, 41] using now the recent NNPDF3.0 NLO global analysis as an input. We focus on the Minimal Supersymmetric extension of the Standard Model (MSSM) with R-parity conservation, where SUSY particles are always pair produced. At a hadron collider such as the LHC, the most copiously produced SUSY particles are expected to be the strongly interacting partners of quarks and antiquarks, the squarks $\tilde{q}$ and anti-squarks $\tilde{q}^*$, and of the gluons, the gluinos $\tilde{g}$. Therefore, in this work we will compute total cross sections for the following processes:

1 $$\begin{aligned} pp \rightarrow \tilde{q}\tilde{q}, \tilde{q}\tilde{q}^*, \tilde{q}\tilde{g}, \tilde{g}\tilde{g}+X, \end{aligned}$$

where by $\tilde{q}(\tilde{q}^*)$ we denote the partners of the massless (anti-) quarks only.

In the present calculations, all flavors of final-state squarks are included and summed over, except top squarks, due to the large mixing effects and the mass splitting in the stop sector. We sum over all light-flavor squarks with both chiralities ($\tilde{q}_{L}$ and $\tilde{q}_{R}$), which are taken to be mass-degenerate. The QCD coupling $\alpha _\mathrm{s}(Q)$ and the parton distribution functions at NLO are defined in the $\overline{\mathrm{MS}}$ scheme with five active flavors. The renormalization and factorization scales are taken to be equal, $\mu =\mu _R=\mu _F$, and a top-quark mass of $m_t=173.21$ GeV is used [73]. The NLO parts of the NLO+NLL predictions are obtained using the public code Prospino [13] while the resummed parts are computed as in Ref. [37]. We refer the reader to [13, 36, 37] for a more thorough discussion of the theory behind the NLO+NLL calculations. For simplicity, in this work we will present results assuming equal squark and gluino masses $m \equiv m_{\tilde{q}}=m_{\tilde{g}}$. In our baseline calculations the factorization and renormalization scales are set equal to the sparticle masses, $\mu =m$.

**Fig. 1:**
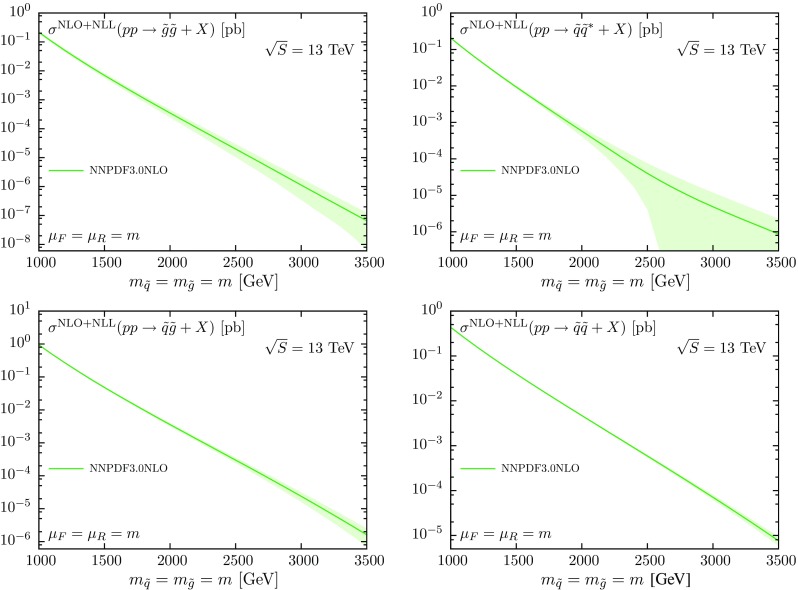
The NLO+NLL total cross sections for the pair production of squarks and gluinos at the LHC $\sqrt{S}=13$ TeV in different channels, obtained using the NNPDF3.0 NLO set. Results are shown as a function of the sparticle mass *m* and include the one-sigma PDF uncertainties

In Fig. 1 we show the NLO+NLL total cross sections obtained using the NNPDF3.0 NLO global fit for the four processes listed in Eq. (1). We present our results, as will be done in the rest of this work, for sparticle masses in the range between 1 and 3.5 TeV. The choice of the lower range is motivated by the upper limits of existing searches of squarks and gluinos by ATLAS [7, 8] and CMS [9] at 8 TeV, while the upper value coincides with the largest sparticle masses that can be probed at the LHC, including its future high-luminosity upgrade [74]. From these absolute cross-section plots we already see that the PDF uncertainties blow up at large masses, reflecting the lack of experimental constraints on the large-*x* PDFs.

It is important to point out that for some specific final states, in particular those driven by the $q\bar{q}$ initial state, for large values of the sparticle mass *m*, roughly for $m\gtrsim 2.5$ TeV, the cross section computed with some replicas of the NNPDF3.0 NLO set might become negative. As discussed in [62], in NNPDF3.0 no ad-hoc conditions are imposed on the shape nor on the positivity of PDFs in the large-*x* region (to avoid any theory bias and the corresponding underestimating of PDF uncertainties), while at the same time one requires the positivity of a number of physical cross sections, such as deep-inelastic structure functions, Drell–Yan rapidity distributions and Higgs production in gluon fusion.

However, it is technically impossible to constrain all possible cross sections to be positive during the fit, and therefore, when for specific processes in extreme regions of the phase space a cross section computed with a NNPDF replica becomes negative, the correct prescription is to set it to zero before evaluating the PDF uncertainty. For the cross sections of Fig. 1, as well as in the rest of the calculations presented in this paper, negative cross sections are always set to zero. We have verified that this has only appreciable effects at very large masses, where PDF uncertainties are huge anyway, and, moreover, that the shift in central values induced by this prescription is always negligible as compared to the intrinsic PDF uncertainty.

In order to illustrate better the size of each of the different effects that enter the calculation, it is often useful to represent the cross sections of Fig. 1 in terms of a *K*-factor by normalizing to the corresponding NLO cross section (with the same input PDF set), that is

2 $$\begin{aligned} K := \frac{\sigma ^{\mathrm {NLO+NLL}}\Big |_{\text {NLO global}}}{\sigma ^{\mathrm {NLO}}\Big |_{\text {NLO global}}}. \end{aligned}$$

**Fig. 2:**
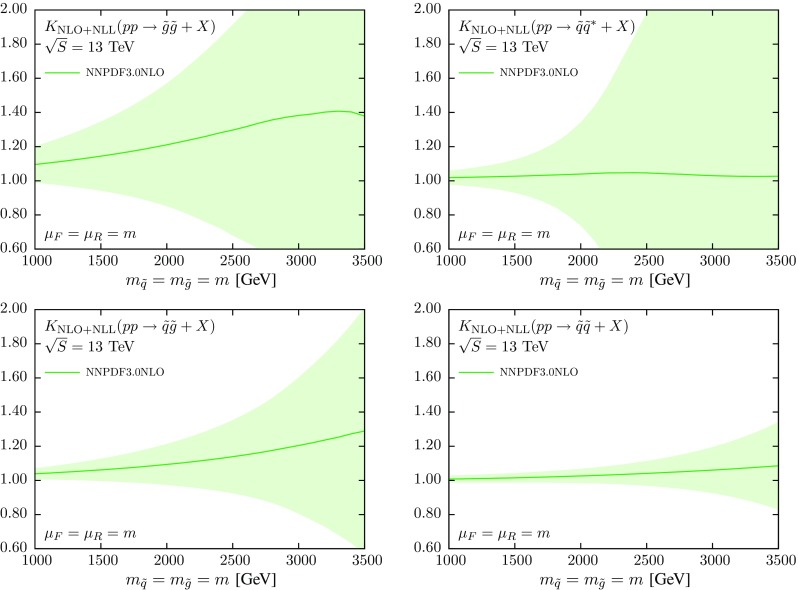
The *K*-factor Eq. (2) between the NLO+NLL and the NLO cross sections, computed in both cases with the NNPDF3.0 NLO global set. We also show the corresponding one-sigma PDF uncertainties

The deviations of this *K*-factor from one indicate the impact of the NLL resummation in the partonic cross sections. These *K*-factors are shown in Fig. 2, where both the NLO+NLL and the NLO cross sections have been computed with the NNPDF3.0 global set. In the computation of Eq. (2), PDF uncertainties are included in the numerator only.

These results quantify the effect of the NLL resummation in the partonic cross sections, for the different initial states and as a function of the sparticle mass *m*. For instance, for the $\tilde{g}\tilde{g}$ final state, NLL resummation leads to an enhancement of the total cross section that increases from 10 % at $m=1$ TeV to 40 % at $m=3$ TeV. For the $\tilde{q}\tilde{g}$ case, resummation increases the NLO cross section by 5 % at 1 TeV and by 20 % at 3 TeV. The effects of threshold resummation are milder for the $\tilde{q}\tilde{q}$ and $\tilde{q}\tilde{q}^*$ final states, where the size of the enhancement is only a few percent. Note in all cases the substantial PDF uncertainties at large masses, specially for the $\tilde{g}\tilde{g}$ and $\tilde{q}\tilde{q}^*$ final states.

**Fig. 3:**
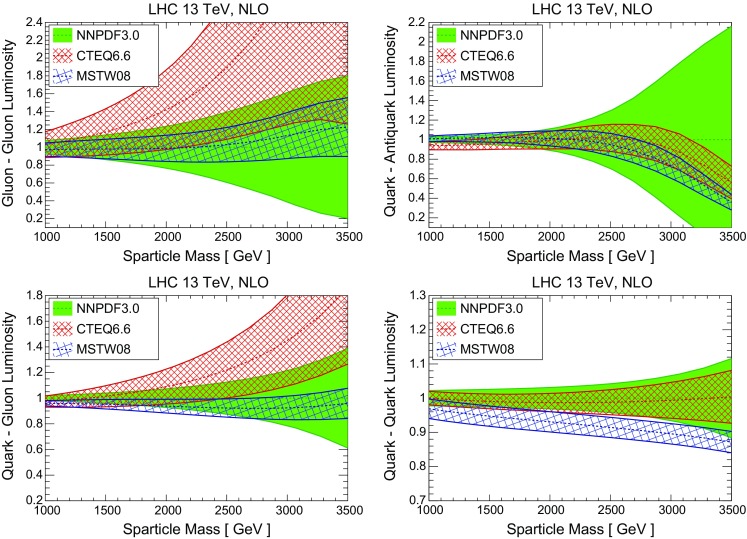
Comparison of PDF luminosities between the NLO NNPDF3.0, CTEQ6.6 and MSTW08 PDF sets, as a function of the sparticle mass *m*. From *top* to *bottom* and from *left* to *right* we show the gluon–gluon, quark–antiquark, quark–gluon, and quark–quark PDF luminosities, normalized to the central value of the NNPDF3.0 result

It is useful to quantify how these updated results for the NLO+NLL cross sections, based on NNPDF3.0 NLO, differ from those previously available, which were computed using the CTEQ6.6 and MSTW08 NLO PDF sets. We study these differences, both for central values and for the total PDF uncertainties, first at the level of PDFs and then at the level of *K*-factors for the resummed cross section. First of all let us compare in Fig. 3 the PDF luminosities between NNPDF3.0, CTEQ6.6 and MSTW08 NLO – this comparison is more useful than that of the individual PDFs, since differences in the PDF luminosities directly translate into differences in the predicted SUSY cross sections.

For the gluon-initiated processes, we find good agreement for the central values of NNPDF3.0 and MSTW08, with the PDF uncertainties of the latter being significantly smaller, especially at high masses. On the other hand, the sizes of the PDF uncertainties in the *gg* and *qg* luminosities are comparable between NNPDF3.0 and CTEQ6.6, with the latter exhibiting a much harder large-*x* gluon: at $m=2.5$ TeV, the *gg* luminosity from CTEQ6.6 is a factor 2 larger than that of NNPDF3.0 (though still consistent within uncertainties). For the *qq* luminosity, there is good agreement between NNPDF3.0 and CTEQ6.6 in terms of both central values and uncertainties, with MSTW08 being systematically smaller by up to 10 %. Finally, for the $q\bar{q}$ luminosity, there is reasonable agreement in the central value of the three sets, but for masses $m\gtrsim 2$ TeV the PDF uncertainty in NNPDF3.0 is much larger than that of the two other PDF sets.

The agreement between NNPDF3.0 and the most recent updates of the CTEQ6.6 and MSTW08 sets, namely CT14 [75] and MMHT14 [76], is improved as compared to what is shown in Fig. 3. In particular now the NNPDF3.0 PDF uncertainty band essentially includes the luminosities from CT14 and MMHT14. Therefore, the results presented in this work would not change substantially if the combination of NNPDF3.0, CT14 and MMHT14 was used instead of only NNPDF3.0 as we do now.

**Fig. 4:**
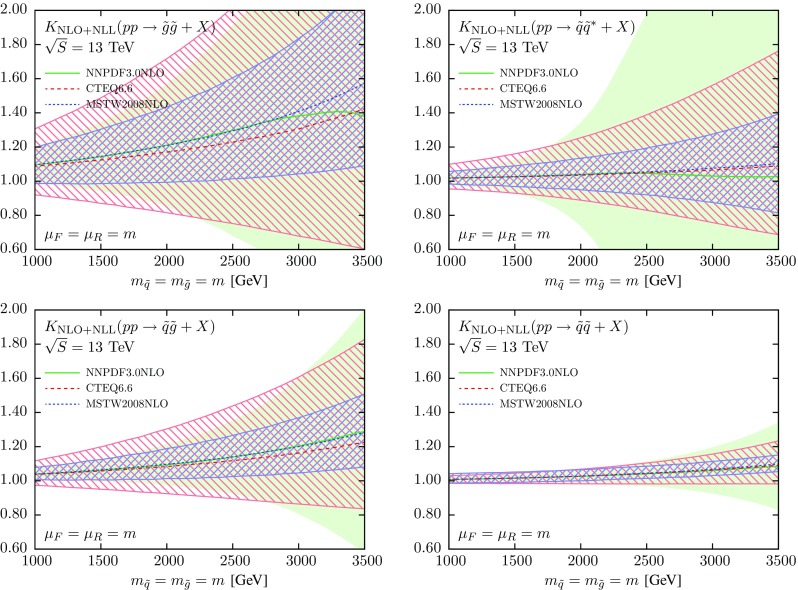
Same as Fig. 2, now including also the results obtained using CTEQ6.6 and MSTW08 NLO, including the corresponding PDF uncertainties in each case

Then in Fig. 4 we show the *K*-factor Eq. (2) between the NLO+NLL and the NLO cross sections, computed with NNPDF3.0, CTEQ6.6 and MSTW08 NLO, with the corresponding PDF uncertainties in each case. In computing the *K*-factor Eq. (2) we use the central member both in the numerator and denominator, but the error members (either replicas or eigenvectors) only in the numerator, to compute the PDF uncertainty. Thus, most of the differences in the central values observed in the luminosity comparison of Fig. 3 will cancel out. On the other hand, the PDF uncertainties at the *K*-factor level should be consistent with those at the PDF luminosity level.

From Fig. 4 we see that for the final states which are predominantly gluon-initiated, $\tilde{g}\tilde{g}$ and $\tilde{q}\tilde{g}$, the size of the PDF error bands is similar between NNPDF3.0 and CTEQ6.6, being somewhat smaller for MSTW08. The central values obtained from the three PDF sets are very similar, except at large values of the sparticle masses *m* where the CTEQ6.6 prediction deviates a bit from that of the other two sets. The size of the PDF uncertainties observed in Fig. 4 is consistent with the comparison of the *gg* and *qg* PDF luminosities in Fig. 3. For quark-initiated processes we find a similar consistency with the PDF uncertainties exhibited by the *qq* and $q\bar{q}$ luminosities. In particular, the size of the PDF uncertainty band for $\tilde{q}\tilde{q}^*$ production is rather larger in NNPDF3.0 as compared to MSTW08 and CTEQ6.6, while for $\tilde{q}\tilde{q}$ production the three PDF sets give similar results.

**Fig. 5:**
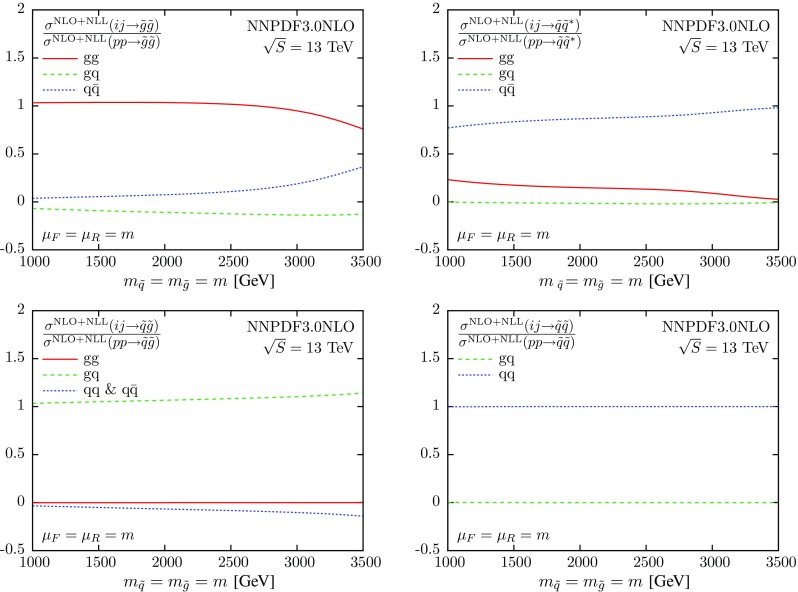
Relative contributions from various initial states to the NLO+NLL squark and gluino cross sections, Eq. (3), calculated with NLO NNPDF3.0 global analysis

It is interesting to understand the results of Figs. 1 and 2 in terms of the decomposition of the initial state into the different components that contribute to the production of each final state. This decomposition is shown in Fig. 5, where we show the ratio

3 $$\begin{aligned} \frac{\sigma ^\mathrm{NLO+NLL}(ij \rightarrow kl)}{\sigma ^\mathrm{NLO+NLL}(pp \rightarrow kl)}, \end{aligned}$$

where *ij* are the different initial-state partonic luminosities, and *kl* label each of the final states.

We observe that the $\tilde{q}\tilde{g}$ and $\tilde{q}\tilde{q}$ final states are produced entirely from the *qg* and *qq* initial states, respectively (these are the only initial states allowed at LO). Therefore, predictions for these two final states will follow closely the behavior of the corresponding PDF luminosities. The $\tilde{g}\tilde{g}$ final state receives contributions both from the $q\bar{q}$ and *gg* initial states, but it is the latter that dominates and determines the behavior of the PDF uncertainty band through the *gg* luminosity; cf. Fig. 3. Similarly, the size and the shape of the PDF uncertainties for $\tilde{q}\tilde{q}^*$ production reflects the properties of the $q\bar{q}$ luminosity as shown in Fig. 3.

To summarize, in this section we have presented updated results for squark and gluino pair production cross sections at NLO+NLL accuracy [37]. So far, available predictions [37, 39–41] were obtained using CTEQ6.6 and MSTW08, while here the recent NNPDF3.0 NLO global fit is used. The combination of an unbiased PDF parametrization with the constraints from all available hard-scattering data, including a number of LHC measurements, make NNPDF3.0 specially suitable to provide a robust estimate of PDF uncertainties for the production of massive particles in the TeV region.

The updated NLO+NLL SUSY cross sections using the NNPDF3.0 NLO global analysis are available from the webpage of the NLL-fast collaboration [77] in the format of fast interpolation grids. These grids provide, for any value of the squark and gluino masses in the range relevant for LHC applications, the NLO and NLO+NLL cross sections together with the overall theory uncertainty, separated in the scale, PDF uncertainties and $\alpha _s$ uncertainties. In the latter case, we assume $\delta \alpha _s=0.0012$ at the 68 % confidence level, and we follow the PDF4LHC prescription [78] for the combination of PDFs and $\alpha _s$ uncertainties for Monte Carlo PDF sets [79].

## Impact of threshold-improved PDFs on the NLO+NLL cross sections

Now we discuss how the NLO+NLL results of the previous section, obtained with the NNPDF3.0 NLO global fit as input, are modified when threshold-improved NLO+NLL PDFs are used instead. The main subtlety here arises from the fact that, as explained in the introduction, the NNPDF3.0 NLO+NLL resummed sets are based on a smaller dataset than their NNPDF3.0 NLO counterparts. Therefore, it is required to devise a prescription for combining results of the fixed-order global fit, which is the most precise in terms of experimental constraints, and the resummed fit, which is based on a more accurate theory but has larger PDF uncertainties. It is also important to emphasize that to assess consistently the impact of NLO+NLL threshold resummation as compared to a NLO fixed-order fit, one should always use PDF sets based on exactly the same input dataset. In the following, using the convention of [63], we will denote by “DIS+DY+top” the NNPDF3.0 fixed-order and resummed fits based on this reduced dataset.

**Fig. 6:**
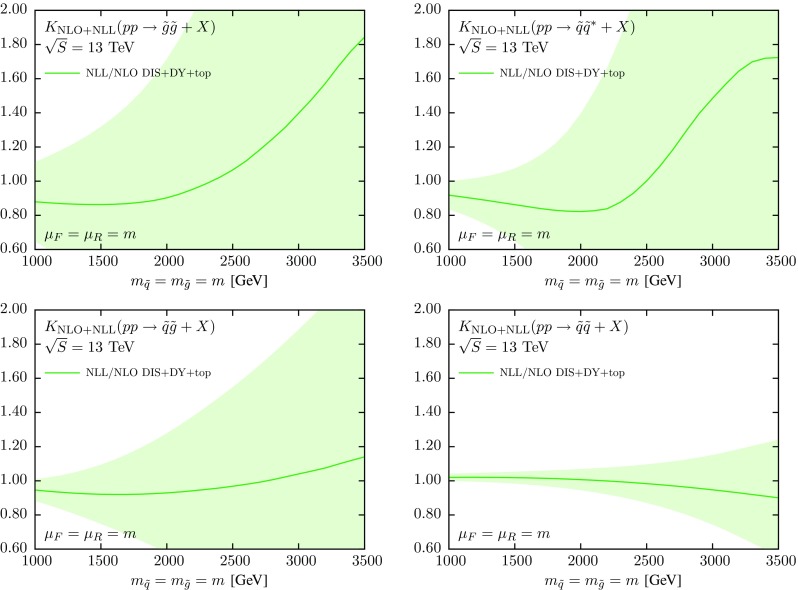
Same as Fig. 2, but now for the *K*-factor defined in Eq. (4)

Now we quantify, using the same settings as in the previous section, the impact of threshold resummation on the supersymmetric particle pair production cross section, with resummation included for partonic cross sections and of parton distributions. This can be achieved by defining a new *K*-factor as follows:

4 $$\begin{aligned} K := \frac{\sigma ^{\mathrm {NLO+NLL}}\Big |_{\text {NLL DIS+DY+top}}}{\sigma ^{\mathrm {NLO}}\Big |_{\text {NLO DIS+DY+top}}}, \end{aligned}$$

where we note the two differences as compared to Eq. (2):

- we use as input the fixed-order and resummed NNPDF3.0 DIS+DY+top fits, rather than the global fits,
- the perturbative order of the PDFs matches that of the partonic cross sections: resummed in the numerator, fixed-order in the denominator.

The results for the *K*-factors defined in Eq. (4) are shown in Fig. 6. As compared to the corresponding results of Fig. 2, obtained using only fixed-order PDFs, there are some important differences. First of all, when a fixed-order PDF is used in the resummed calculation, Fig. 2, we found that the effect of threshold resummation was almost always to increase the total cross section monotonically as a function of the sparticle mass. The only exception was for very high masses for $\tilde{g}\tilde{g}$ and $\tilde{q}\tilde{q}^*$, where PDF uncertainties are very large and the central value of the prediction is affected by, respectively, large fluctuations. On the other hand, when threshold-improved PDFs are used together with resummed cross sections, Fig. 6, the results are qualitatively different.

For sparticle masses below 2 TeV we now find a *K*-factor smaller than unity, except for the $\tilde{q}\tilde{q}$ final state. The absolute size of the *K*-factors for $m\le 2$ TeV is around 0.9 for $\tilde{g}\tilde{g}$ and $\tilde{q}\tilde{g}$, between 0.8 and 0.9 for $\tilde{q}\tilde{q}^*$, and a few percent above unity for $\tilde{q}\tilde{q}$. This behavior changes at larger sparticle masses, where the *K*-factors can become much larger, specially for the $\tilde{g}\tilde{g}$ and $\tilde{q}\tilde{q}^*$ final states. The origin for this turnover is the impact of the resummation in the partonic cross sections, which becomes dominant and compensates the suppression due to the resummation on the PDFs. In any case, PDF uncertainties are very large in this region, above 2 TeV.

**Fig. 7:**
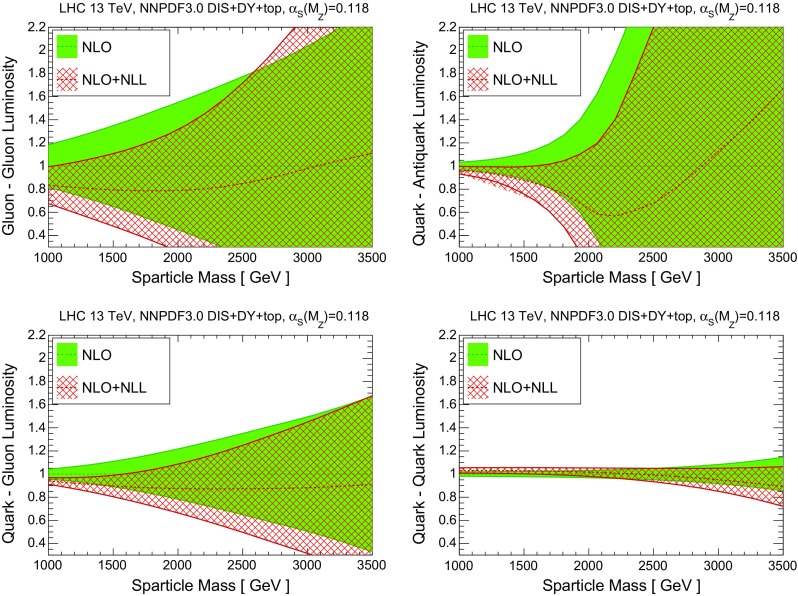
Comparison of PDF luminosities between the fixed-order NLO and resummed NLO+NLL NNPDF3.0 DIS+DY+top fits, as a function of the produced sparticle mass, in the same range as those of the cross sections in Fig. 6. From *top* to *bottom* and from *left* to *right* we show the gluon–gluon, quark–antiquark, quark–gluon and quark–quark PDF luminosities, normalized to the central value of the NLO fit

In order to understand these results, it is useful to compare the DIS+DY+top NLO fixed-order and NLO+NLL resummed fits in terms of partonic luminosities, using exactly the same range of invariant masses as that shown in Fig. 6. The results are shown in Fig. 7. Taking into account the initial PDF combinations that contribute to each final state, it is possible to quantitatively understand various interesting features. For example, we see that the *gg* luminosity is suppressed due to resummation by about 20 % for masses *m* between 1 and 2.5 TeV, and then it becomes essentially the same as the fixed-order counterpart within the very large PDF errors. Correspondingly, towards the lower end of the considered sparticle mass spectrum, the *K*-factor for $\tilde{g}\tilde{g}$ cross section in Fig. 6 is smaller than in Fig. 2 by around ${\sim } 15~\%$, and then it starts to grow as *m* is increased when the resummation in the partonic cross sections starts to dominate.

Therefore, we conclude that the effect of using threshold-improved PDFs in a resummed calculation cannot be neglected, and is important to take into account, since it modifies both the qualitative and the quantitative behavior of the high-mass sparticle pair production cross sections at the NLO+NLL accuracy. However, the *K*-factors of Fig. 6 cannot be taken as our best results, since they are affected by much larger PDF uncertainties as compared to the global NNPDF3.0 fit results; cf. Figs. 3 and 7. Therefore, we need a prescription to include the effect on the SUSY cross sections of the threshold resummation in the PDFs, while keeping all the experimental information on large-*x* PDFs available in the NNPDF3.0 global fit.

In this respect, we have explored a number of prescriptions to combine the global (fixed-order) and DIS+DY+top (resummed) results for the sparticle cross sections. Here we will show results for two possible prescriptions. The first is defined as follows:

5 $$\begin{aligned} K := \frac{ \sigma ^{\mathrm {NLO+NLL}}\Big |_{\text {NLO global}} }{ \sigma ^{\mathrm {NLO}}\Big |_{\text {NLO global}} } \times \frac{\sigma ^{\mathrm {NLO+NLL}}\Big |_{\text {NLL DIS+DY+top}} }{ \sigma ^{\mathrm {NLO+NLL}}\Big |_{\text {NLO DIS+DY+top}}}.\nonumber \\ \end{aligned}$$

It amounts to an overall rescaling of the *K*-factor Eq. (2), the result obtained using the global NLO set as input, by a factor that accounts for the differences in the NLO+NLL calculation when using either NLO+NLL or NLO PDFs as input. Note that in the limit in which the dataset of the DIS+DY+top fit would become identical to that of the global (when all observables in the global fit can be simultaneously resummed), this definition automatically reduces to the result which would be obtained using a NLO+NLL global fit in the numerator and a NLO global fit in the denominator, i.e.,

6 $$\begin{aligned} K := \frac{ \sigma ^{\mathrm {NLO+NLL}}\Big |_{\text {NLL global}} }{ \sigma ^{\mathrm {NLO}}\Big |_{\text {NLO global}} }. \end{aligned}$$

Note that in Eq. (5) PDF uncertainties are not included; we are only interested in quantifying the shift in the central value of the NLO+NLL cross sections when resummed (rather than fixed-order) PDFs are used as input to the calculation.

An improvement with respect to the prescription of Eq. (5) can be achieved by rescaling each initial state separately, that is, the overall *K*-factor is now defined as

7 $$\begin{aligned} K = \frac{\sigma ^{\mathrm {NLO+NLL}}_{qq}\Big |_{\text {rescaled}}+\sigma ^{\mathrm {NLO+NLL}}_{qg}\Big |_{\text {rescaled}}+\sigma ^{\mathrm {NLO+NLL}}_{gg}\Big |_{\text {rescaled}}}{\sigma ^{\mathrm {NLO}}\Big |_{\text {NLO global}}}\nonumber \\ \end{aligned}$$

where we have defined the *rescaled* cross sections for the various initial states as follows:

8 $$\begin{aligned} \sigma _{ij}^{\mathrm {NLO+NLL}}\Big |_{\text {rescaled}}= & {} \sigma _{ij}^{\mathrm {NLO+NLL}}\Big |_{\text {NLO global}}\nonumber \\&\times \frac{\sigma _{ij}^{\mathrm {NLO+NLL}}\Big |_{\text {NLL DIS+DY+top}}}{\sigma _{ij}^{\mathrm {NLO+NLL}}\Big |_{\text {NLO DIS+DY+top}}}. \end{aligned}$$

The motivation for this second prescription Eq. (7) is that it might be more accurate to include the effect of resummation in the PDFs separately in each of the individual partonic channels. As we will now show, from the practical point of view the two prescriptions Eqs. (5) and (7) yield very similar numerical results, which indicates that our proposed strategy is robust.

**Fig. 8:**
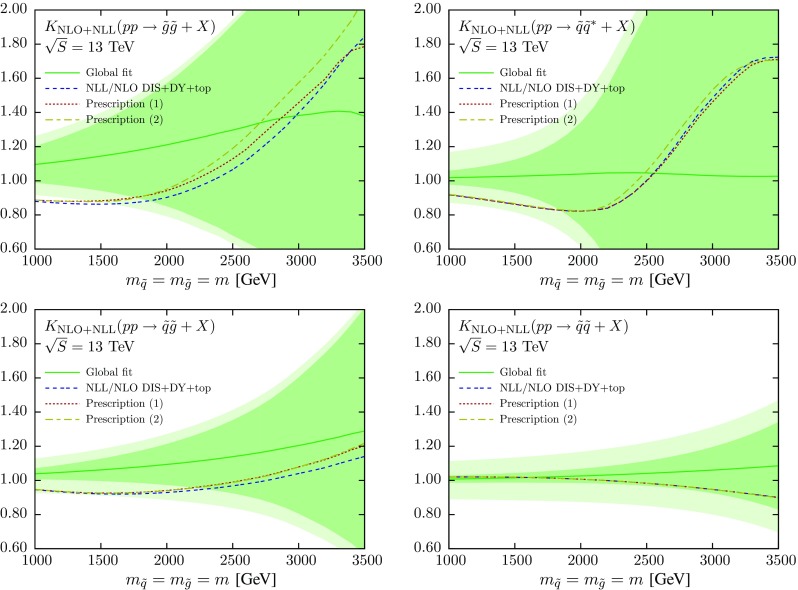
Comparison of the NLO+NLL *K*-factors obtained using the NNPDF3.0 NLO global fit, Eq. (2) with the corresponding *K*-factors obtained accounting for the effect of resummation in the input PDFs, Eq. (5), called here Prescription (1), and Eq. (7), called here Prescription (2). In the case of the global fit, we show the total uncertainty band (*light green band*) as well as the PDF-only uncertainty band (*solid green band*). We also show the *K*-factor Eq. (4) determined from the DIS+DY+top fit

Using these two prescriptions, we can now compare the results obtained using as an input the global NNPDF3.0 NLO fit, Fig. 2, with those obtained using the threshold-improved PDFs. This comparison is shown in Fig. 8, where we first show the resummed *K*-factors obtained using NNPDF3.0NLO, Eq. (2), together with the associated total theory uncertainty band. For the latter, we separate the PDF-only uncertainty (solid band) from the total theory error band (lighter band), where PDF uncertainties have been added linearly to scale uncertainties. The scale error is estimated by varying simultaneously the factorization and renormalization scales up and down by a factor two with respect to their reference value $\mu _R=\mu _F=m$, the sparticle mass. We then show the corresponding *K*-factors obtained when accounting for the effect of resummation in the input PDFs, Eqs. (5) and Eq. (7), using the two different prescriptions for the rescaling. For completeness, we also include the *K*-factor obtained from Eq. (4) determined from the DIS+DY+top fit.

First of all, we note that the choice of prescription has a very small effect, except in the region where the PDF uncertainties are huge. For all final states in most of the relevant sparticle mass region, the total theory uncertainty band encompasses the shift in the central value induced by the use of resummed PDFs, as compared to the global fixed-order fit result. The only exception is $\tilde{g}\tilde{g}$ production for $m\le 1.5$ TeV, where the agreement is only marginal. We also see that as we increase the sparticle mass, PDF uncertainties dominate over scale uncertainties (which are roughly independent of *m*), in some cases by a large amount. For the $\tilde{g}\tilde{g}$, $\tilde{q}\tilde{q}^*$, $\tilde{q}\tilde{q}$, and $\tilde{q}\tilde{g}$ final states we find that PDF uncertainties begin to dominate over scale uncertainties for $m\ge$ 1, 1.5, 2.5 and 1.5 TeV, respectively.

It is worth emphasizing that several of the features of how the cross section *K*-factors in Fig. 8 are modified due to the use of resummed PDFs can be directly traced back to the corresponding modifications at the level of PDF luminosities, as illustrated in Fig. 7. For instance, the turnover in the *K*-factor for $\tilde{q}\tilde{q}^*$ production from $K<1$ to $K>1$ around $m\simeq 2.5$ TeV can also be observed for the central value of the $q\bar{q}$ luminosity. This behavior can, in turn, be traced back to two facts. First of all, as quantified in Fig. 5, each of the SUSY final states considered here, $\tilde{q}\tilde{q}$, $\tilde{q}\tilde{q}^*$, $\tilde{q}\tilde{g}$, and $\tilde{g}\tilde{g}$, has a dominant partonic production channel, *qq*, $q\bar{q}$, *qg*, and *gg*, respectively. Second, the ratio of the *K*-factors in Eqs. (4) and (2) is approximately given by the ratio of NLO+NLL cross sections computed using resummed and fixed-order DIS+DY+top NNPDFs, while the relation is exact for the ratio of the *K*-factors in Eqs. (5) and (2). This also explains why in Fig. 8 the *K*-factors obtained using prescriptions are very close to *K*-factors constructed from Eq. (4).

Figure 8 is the main result of this work: for the first time we have performed a NLO+NLL calculation of supersymmetric particle pair production at hadron colliders accounting for the effects of threshold resummation both in the partonic cross sections and in the PDFs. As compared to the results obtained using the global NNPDF3.0NLO fit as input, we find that including the effect of resummation in the PDFs modifies the resummed NLL *K*-factor both in a qualitative and in a quantitative way. This shift is, however, contained within the total theory uncertainty band of the NNPDF3.0NLO result, and therefore the use of threshold-resummed PDFs does not modify the current SUSY exclusion bounds.

Similarly to the behavior of the NLL K-factor, it can be shown that the modification of the NNLL K-factor will be mostly driven by the differences between the NNLL and NNLO PDF luminosities obtained on the basis of DIS+DY+top fits. Given that the global NNLL K-factors follow the behavior of the NLL K-factors [44, 46] with the NNLL corrections in general smaller than NLL, and that the impact of threshold resummation in PDF analysis at NNLO appears to be much less than at NLO [63], we believe our conclusions regarding the behavior of the K-factor will not change dramatically after increasing the accuracy to NNLL.

## Summary and outlook

In this work we have presented updated NLO+NLL predictions for squark and gluino pair production at the LHC Run II obtained using the NNPDF3.0 NLO global fit. Our calculations are based on fixed-order NLO partonic cross sections matched to NLL threshold resummation. We have then studied the impact in the calculation of using threshold-improved PDFs together with the resummed partonic cross sections, finding that both the quantitative and the qualitative behavior of the NLO+NLL cross sections is modified. However, we also find that the shift induced by the resummed PDFs is contained within the total theory uncertainty band of the standard calculation.

Given that PDFs with threshold resummation are still in their infancy, and that the shift they induce is within the total theory error band of the calculation using as input NNPDF3.0 NLO global fit, we prefer to still adopt the latter in our reference calculations. This choice is reasonable for the time being in order to determine exclusion limits from the searches for supersymmetry at the LHC, though it would become inadequate in the case of a discovery of supersymmetric particles in the next years.

The main limitation of the NNPDF3.0 threshold-resummed sets is the fact that they are based on a reduced dataset. This forces us to introduce somewhat ad-hoc prescriptions to combine them with the global fit results. In order to improve the situation, and to bypass the need of the prescriptions (and being able to use the resummed PDFs as central value in our calculation), it should be important to produce truly global versions of the resummed fits of Ref. [63]. This requires in particular the availability of NLL and NNLL calculations for inclusive jet production in a format ready to use.

Another important message from our study is the role of PDF uncertainties in high-mass sparticle pair production. PDF errors are the dominant source of theoretical uncertainty: in the case of the discovery of sparticles in the TeV region at Run II, it would be difficult to accurately pin down their properties unless one is able to reduce these PDF uncertainties. Fortunately, it is possible to use the LHC data itself [80] as input to the global fit to better constrain the large-*x* PDFs that drive the PDF uncertainties in the high-mass region. Examples of measurements that should be helpful in this respect are top-quark pair production, both for inclusive cross sections [81], and for differential distributions [82], high-mass Drell–Yan production [83–85] and inclusive jet and dijet production [86, 87]. For all these processes, measurements from ATLAS, CMS and LHCb at Run I are already available, and more precise data from Run II will soon extend their kinematical coverage well into the TeV region.

In addition to the phenomenology, it should be emphasized that NLO supersymmetric pair production provides a highly non-trivial theoretical laboratory to test the perturbative convergence of perturbative QCD calculations. In this respect, the availability for the first time of threshold-improved PDFs provides a unique opportunity to test in detail the interplay of the effects of higher-order resummation in the various pieces of the calculation.

The updated NLO+NLL squark and gluino production cross sections with the NNPDF3.0NLO global fit are available in the NLL-fast format [77]. In addition, the cross sections obtained in this work using the threshold-resummed PDFs are also available from the authors upon request.

## Appendix A: Threshold-resummed PDFs from a scheme transformation

The threshold-improved PDFs of Ref. [63] incorporate, though the global fitting procedure, the large logarithms of the cross sections involved in the fit to all orders. Such logarithms are universal at the leading logarithmic level, and to a considerable extent at the next-to-leading level and beyond as well. This incorporation is then numerical in nature, and indeed, as we observe in this paper, corrections for other cross sections are reduced, because a large part of the resummed threshold logarithms in the higher corrections now reside in the PDFs. In this appendix we point out another method by which one might include large threshold logarithms in the PDFs, using a dedicated mass factorization scheme choice.

In general one may represent mass factorization of initial-state collinear divergences for PDFs in the following schematic way:

9 $$\begin{aligned} \hat{f}_i = \Gamma _{ik,\mathrm {scheme}} (\alpha _s(\mu ),Q^2,\mu ^2,\varepsilon )\otimes f_k(\mu ), \end{aligned}$$

where *i*, *k* label parton flavors, $\hat{f}_i$ ($f_i$) are bare (factorized) PDFs, $\mu$ is the factorization scale, and *Q* a representative physical scale for the process. The functions $\Gamma _{ik,{\mathrm {scheme}}}$ are known as *transition functions*, and they take the general form

10 $$\begin{aligned}&\Gamma _{ik,{\mathrm {scheme}}} (\alpha _s(\mu ),Q^2,\mu ^2,\varepsilon )(z) = \delta (1-z)\delta _{ik}\nonumber \\&\quad +\,\frac{\alpha _s(\mu )}{8\pi }\left( \frac{-1}{\varepsilon } +\gamma _E - \ln 4\pi \right) P_{ik}(z) +\cdots \nonumber \\&\quad +\,f_{ik} (\alpha _s(\mu ),Q^2,\mu ^2,z), \end{aligned}$$

where the ellipses indicate higher-order pole terms, and the expression is in $$4-2\varepsilon $$ dimensions. The choice of finite terms $$f_{ik}$$ then determines the factorization scheme. In Eq. (10) $$ P_{ik}(z)$$ are the standard DGLAP splitting functions.

In the following, we set $\mu ^2=Q^2$ in these functions, and we drop the corresponding arguments. One may choose the $f_{ik}$ according to specific preference, provided that the charge and momentum sum rules for the PDFs so defined remain satisfied. For instance, for the momentum sum rule the following conditions must hold:

11 $$\begin{aligned} \begin{aligned}&\int \mathrm{d}z\, z\, \left( \Gamma _{gg} + 2n_f \Gamma _{qg} \right) =1,\\&\int \mathrm{d}z\, z\, \left( \Gamma ^S_{qq} + \Gamma _{gq} \right) =1, \end{aligned} \end{aligned}$$

where the superscript *S* indicated the singlet combination of quark PDFs (sum over all active quarks and antiquarks), and $n_f$ is the number of active quark flavors.

All modern PDF sets are defined in the $\overline{\mathrm {MS}}$ scheme, for which one has

12 $$\begin{aligned} f_{ik} = 0. \end{aligned}$$

The advantages of this scheme are first process independence, and second, ease of implementation. Note that due to the properties of the splitting functions the charge and momentum sum rules (11) are automatically preserved. In the past also the DIS scheme has been used. In this scheme one chooses the $f_{ik}$ functions such that the non-singlet DIS quark coefficient is equal to its lowest order expression, to any order in perturbation theory. Besides the DIS scheme, also a Drell–Yan scheme has been proposed in [88], for the computation of inclusive $W+\gamma$ production at NLO.

One could also define a variant of this scheme, the threshold-resummation (TR) scheme, in which e.g. the Drell–Yan and Higgs cross sections are defined to be equal to their lowest order cross sections, and the *qg* and $\bar{q}g$ transition functions are defined such that the sum rules Eq. (11) are satisfied. In such a TR scheme, when fitting to data, the threshold logarithms are largely subtracted from the partonic cross sections in the fit, by design, and absorbed into the TR scheme PDFs. To use such TR scheme PDFs for other partonic cross sections one must of course subtract the $f_{ik}$ functions for those cross sections as well. If these cross sections are also threshold resummed, a large amount of cancelation should occur through that subtraction.

Thus, for the TR scheme one might choose the Drell–Yan and the Higgs cross section

13 $$\begin{aligned} f_{qq}(z) = f_{\bar{q}\bar{q}}(z)= \frac{1}{2} \sigma _{q\bar{q}}^{\mathrm {DY,N^iLL}}(z), \end{aligned}$$

and

14 $$\begin{aligned} f_{gg}(z) = \frac{1}{2} \sigma _{gg}^{H,\mathrm {N^iLL}}(z). \end{aligned}$$

For the latter in particular one might also consider the inclusive top cross section, which is dominated by the gluon distribution as well, but its color structure is more involved than Higgs production. Whether one takes on the right hand side of Eqs. (13) and (14) only the logarithmic terms or uses the full matched resummed cross section, including hard part, is a matter of choice.

The advantage of such a TR factorization scheme would be that it is analytical, allowing for better insight into its effects. The downside, however, is clearly that substantial additional work would be required for users of such TR scheme PDFs, who must convert their partonic cross sections to this scheme. Moreover, a TR scheme method would be more constraining because the sum rules (11) must be obeyed, so any changes in subtraction terms are highly constrained. For instance, the off-diagonal transition functions $\Gamma _{qg}$ and $\Gamma _{gq}$ also incorporate leading logarithmic effects, via the sum rules.

In the approach of [63], compliance of Eq. (11) is ensured in the fitting procedure, and it holds whatever cross sections one chooses to include in the fit. Therefore, while a more extended study of such a TR scheme approach might be interesting, employing the framework of [63] in this paper is advantageous since it achieves the same goals with greater flexibility.
